# Memory integration in the autobiographical narratives of individuals with autism

**DOI:** 10.3389/fnhum.2015.00076

**Published:** 2015-02-13

**Authors:** Rachel S. Brezis

**Affiliations:** Sagol Center for Applied Neuroscience, School of Psychology, Interdisciplinary CenterHerzliya, Israel

**Keywords:** autism spectrum disorder, autobiographical memory, episodic memory, integrative functions, self, personal narratives, medial prefrontal cortex, memory specificity

## Introduction

As part of a unifying theory of autism, Ben Shalom (2009) proposed that while procedural, perceptual and semantic memory functions are intact in Autism Spectrum Disorder (ASD), the more integrative level of episodic memory is impaired. According to Ben Shalom, this reduced integration may be due to the reduced function of the medial prefrontal cortex (mPFC), which may also explain the reduced integration found in motor, sensory-perceptual and emotional processes in ASD. The present review examines this hypothesis, by focusing on evidence regarding autobiographical memory (AM) episodes in ASD—arguably the highest form of memory integration processes.

Most research on memory in ASD thus far has focused on memory for experimentally-presented stimuli (Lind, 2010; Boucher et al., 2012). The present paper builds on this literature to examine the rich evidence that has recently accumulated from in-depth, systematic studies of AM in ASD—memories of personally-related events that are naturalistically accumulated over a person's lifetime. Of note, research on AM is limited in its focus on memories that cannot be as readily verified (but see Bruck et al., 2007), and in its reliance on high-functioning verbal individuals. Nonetheless, studies of AM provide us with an unparalleled perspective on the naturalistic process of memory integration in ASD. Specifically, this review aims to determine how well memory episodes are integrated in ASD; which elements become integrated and which do not; whether the ability to form integrated, episodic memories relates to other cognitive and emotional capacities; and how this pattern of integration changes over time.

### Semantic and episodic autobiographical memory (AM)

The declarative memory system comprises semantic and episodic components. Semantic memories are memories of timeless, de-contextualized facts. Episodic memory refers to personal events recollected in the context of a particular time and place, with some reference to oneself as a participant in the episode (Tulving, 2002). Thus, episodic memories involve two functions: the ability to bind different perceptual elements; and, in humans, the ability to perceive of oneself within this context. On a neurobiological level, episodic memory storage and retrieval are thought to involve the interaction of cortical association areas, in which basic sensory information regarding what occurred and where is stored; the hippocampus, which binds these elements into cohesive memories of individual events; and the mPFC, which further contextualizes these events into schemas, such as the self (Preston and Eichenbaum, 2013).

AM refers to memory for information pertaining to the self; and while it is often viewed as overlapping with episodic memory, the two are not synonymous (Gilboa, 2004). Episodic memory is a memory system, while AM is a type of content (Gardiner, 2008). Thus, episodic memory functions can encompass both AM and simple phenomena that do not necessarily represent self-relevant information (e.g., source memory). At the same time, AM in fact comprises of both semantic and episodic knowledge (e.g., semantic knowledge of one's date of birth, alongside an episodic memory of one's last birthday).

In children with ASD, both semantic and episodic AM is reduced (Bruck et al., 2007; Bon et al., 2012; Goddard et al., 2014), though by adulthood, adults with ASD show a spared memory for semantic AM, alongside reduced episodic AM (Klein et al., 1999; Crane and Goddard, 2008). These studies suggest that as semantic AM may grow in ASD, episodic AM impairments are pervasive. These results fit with the general memory profile in ASD, viz., spared semantic memory alongside difficulties with episodic memory, which is found across experimental studies (Boucher and Bowler, 2008). The present review concerns itself primarily with episodic AM in ASD, though semantic memory will be discussed as it relates to the content of autobiographical narratives.

### Episodic specificity and narrative integration

The most common marker of successful episodic AM integration is its degree of specificity: to what extent is the memory vivid? Memory specificity is considered a marker of hippocampus and mPFC re-engagement during memory retrieval (Piolino et al., 2009). Memories are coded as “specific” if, in response to a cue, participants provide a narrative that is specific in time and place (e.g., “on my last birthday I went to Yogurtland with friends”); rather than a vague or repetitive occurrence. Using several variants of this task, autobiographical narratives in ASD have consistently been shown to be reduced in specificity, compared with control participants, in every reported study (Goddard et al., 2007, 2014; Crane and Goddard, 2008; Crane et al., 2009, 2010, 2012, 2013; Tanweer et al., 2010; Brezis et al., 2012; Chaput et al., 2013; Maister et al., 2013). In some cases, AM retrieval in individuals with ASD than is also slower and more effortful than for control participants (Goddard et al., 2007; Chaput et al., 2013).

More broadly, the narrative structure of AM in both children and adults with ASD has been found to be reduced in integration. The personal narratives of adults with ASD are less likely to have an organizing high-point and resolution (McCabe et al., 2013); and those of children with ASD are more likely to resemble a list of actions or descriptions than a goal-directed sequence (Goldman, 2008). Furthermore, both children and adults with ASD employ fewer causal connectors and evaluations and less complex syntax in their personal narratives than TD controls (Losh and Capps, 2003, 2006; King et al., 2013; McCabe et al., 2013). Together, these studies point to a general impairment of AM integration in ASD, manifest both in reduced specificity and narrative structure.

### Memory content

The richness of autobiographical narrative data allows an examination not just of what is missing from the personal memories of individuals with autism, but also of what they include. Early studies found that both the published and experimentally-induced autobiographical narratives of adults with autism included very concrete, visually-oriented reports (Hurlburt et al., 1994; Frith and Happé, 1999); and this finding was further replicated in a study of children's narratives (Losh and Capps, 2006; but see Boucher, 2007 and Ben Shalom et al., 2010 for a conflicting case study). Indeed, these findings fit with Ben Shalom's (2009) claim that perceptual memory, presumably subserved by a network including the rhinal cortex, is unimpaired in autism.

Further studies of the *content* of personal memories in ASD found striking differences in the topics raised by individuals with ASD and TD controls. For instance, youth with ASD are less likely to mention humans (e.g., family members), and more likely to mention non-humans, than TD youth (Brezis et al., 2012; Chaput et al., 2013). Given that difficulties in social-emotional processing are considered a core symptom of ASD, it is not surprising that individuals with autism have a reduced memory for social and emotional content (Souchay et al., 2013; Brezis et al., 2014). Beyond their reduced mentions of self and others, children and adults with ASD consistently make fewer evaluations regarding their own, or others' mental states (Losh and Capps, 2003, 2006; Brezis et al., 2012; Brown et al., 2012; Bang et al., 2013; King et al., 2013).

The reduced focus on self and others and increased focus on fictional characters in the personal narratives of individuals with autism is echoed in naturalistic, ethnographic studies of autism. During dinnertime conversations, youth with autism are more likely to spontaneously recount a pre-existing narrative they read or viewed, than a personal event they experienced, compared with their TD interlocutors (Solomon, 2004). Furthermore, ASD individuals' personal interests in finance, dinosaurs, and religious narratives, or even their tendency to hoard, can become woven into their identities and sense of self (Nickrenz, 2007; Sirota, 2010; Brezis, 2012; Skirrow et al., 2014). Returning to Ben Shalom's hypothesis, spared semantic memory in individuals with high-functioning ASD may indeed serve them as a compensatory mechanism for episodic AM. Further research is needed to understand the ways in which semantic knowledge becomes integrated into their personal memories and identities.

### Cognitive correlates of episodic AM

Different cognitive functions have been hypothesized to affect AM patterns in ASD. Impairments in executive functions in ASD, viz., the ability to bind and integrate information (Bowler et al., 2014), correlate significantly with AM performance (Maister et al., 2013; Goddard et al., 2014). Further, AM ability is related to Theory of Mind (Adler et al., 2010; Crane et al., 2013) and emotional understanding (Losh and Capps, 2003); presumably because the ability to understand others' thoughts and feelings may help one understand that one's own experiences change over time. Emergent findings point to a strong relation between difficulties in AM and future thought in ASD, which may be subserved by a common difficulty in binding and constructing mental events (Lind et al., 2014).

Other basic functions of memory, such as visual, verbal and working memory are not associated with AM (Crane et al., 2013; Goddard et al., 2014). And while reduced AM is considered a symptom of depression, depressed mood is not associated with AM in ASD (Crane et al., 2013). Further, though language ability relates to performance on AM tasks (King et al., 2013), difficulties in AM appear to extend beyond language difficulties (Losh and Capps, 2003; Lind et al., 2014).

The relation between AM and self-understanding is more complex. In line with known atypicalities in self-knowledge and self-awareness in ASD (Lind, 2010; Tanweer et al., 2010), adults with ASD have been found to extract less meaning from their personal memories (Crane et al., 2010), and were less likely to organize their self-related memories around the self (Crane et al., 2009). These findings suggest a failure to use the self as an effective memory organizational system in ASD. Further research should clarify whether reduced self-concept leads to reduced AM or results from it (Lind et al., 2014).

### Episodic AM for recent and remote events

Examining episodic AM for different periods in participants' lifetimes, studies have found that AM in ASD follows typical patterns of memory deterioration—with more specific memories for recent than remote events—in both adults (Crane and Goddard, 2008; Tanweer et al., 2010) and children (Bruck et al., 2007; Goddard et al., 2014; but see Bon et al., 2012). Interestingly, Crane and Goddard (2008) found that adults with ASD lack the typical increase of memories around adolescence years (the “reminiscence bump”) that is associated with identity-building memories. Further cross-sectional and longitudinal research is needed to replicate and extend these findings.

## Conclusions

According to Tulving (2002), episodic memory is a “late-developing, and early-deteriorating past-oriented memory system, more vulnerable than other memory systems to neuronal dysfunction” (p. 5). Thus, it is perhaps not surprising that in autism, episodic AM is reduced in specificity and structural integration. Nevertheless, an examination of the autobiographical narratives of individuals reveals certain unique characteristics that are shaped by their specific difficulties with social-emotional processing and integration, possibly pointing to a differently configured memory system that may require its own descriptive terminology (Mottron et al., 2008). The narratives of individuals with ASD tend to focus on perceptual or semantic details, favoring pre-structured narratives over lived, emotional experiences. And AM impairments are related to underlying difficulties with integration and emotional understanding, but are not consistently related to other psychological functions, such as depressed mood. As our understanding of AM in ASD deepens, it will be necessary to further delineate the ways in which the memory profile of ASD overlaps and differs from that of other neurological conditions (such as depression, amnesia and other developmental disorders), in order to identify unique and common processes of memory in ASD.

The rich findings regarding AM in ASD call for several additional avenues of research. First, while reduced episodic AM integration suggests atypical engagement of the hippocampus and mPFC during memory processes (Ben Shalom, 2009), and several studies have shown reduced mPFC engagement during self-related processes in ASD (see Uddin, 2011 for a review), no neurobiological study to date has directly examined AM in ASD. Second, it is necessary to further examine the complex relation between AM, self-understanding and identity, both through careful experimental research that disentangles these components, and through further naturalistic studies that seek to determine how they are *integrated*. Finally, more longitudinal research, especially around the “reminiscence bump” in adolescence, is needed in order to track the developmental trajectory of semantic and episodic AM in ASD (Bon et al., 2012). Together, these studies will enable us to develop subtle interventions to strengthen the memory integration of individuals with autism, while maintaining their unique characteristics and supporting their self-identity.

### Conflict of interest statement

The author declares that the research was conducted in the absence of any commercial or financial relationships that could be construed as a potential conflict of interest.

## Abbreviations

AM: Autobiographical Memory
ASD: Autism Spectrum Disorders
AS: Asperger Syndrome
mPFC: medial Prefrontal Cortex
TD: Typically Developing.
